# Umbilical cord plasma concentrate has beneficial effects on DNA methylation GrimAge and human clinical biomarkers

**DOI:** 10.1111/acel.13696

**Published:** 2022-09-02

**Authors:** James Clement, Qi Yan, Megha Agrawal, Ramon E. Coronado, John A. Sturges, Markus Horvath, Ake T. Lu, Robert T. Brooke, Steve Horvath

**Affiliations:** ^1^ Betterhumans Inc. Gainesville Florida USA; ^2^ Department of Chemical, Pharmaceutical and Agricultural Sciences University of Ferrara Ferrara Italy; ^3^ Epigenetic Clock Development Foundation Torrance California USA; ^4^ Transplant Department, UT Health San Antonio San Antonio Texas USA; ^5^ Department of Obstetrics and Gynecology Baylor College of Medicine Houston Texas USA; ^6^ Signature Biologics Irving Texas USA; ^7^ Crown Scientific San Antonio Texas USA; ^8^ John A Sturges, M.D. Coeur d'Alene Idaho USA; ^9^ Department of Human Genetics, David Geffen School of Medicine University of California Los Angeles California USA; ^10^ Altos Labs San Diego USA; ^11^ Department of Biostatistics, Fielding School of Public Health University of California Los Angeles California USA

**Keywords:** clinical trial, epigenetic clocks, exosome treatment, umbilical cord plasma, young plasma

## Abstract

Plasma transfusions are standard treatments to replace missing proteins in people with rare genetic diseases. Prior studies have demonstrated that heterochronic parabiosis has beneficial effects on several tissues of old animals receiving young blood. Human clinical trials are currently underway to investigate whether the infusion of plasma or plasma‐derived factors from young donors can be used to mitigate human age‐related conditions. Here, we use data from a safety study (*n* = 18, mean age 74) to investigate whether human umbilical cord plasma concentrate (hereinafter Plasma Concentrate) injected weekly (1 ml intramuscular) into elderly human subjects over a 10‐week period affects different biomarkers, including epigenetic age measures, standard clinical biomarkers of organ dysfunction, mitochondrial DNA copy number (mtDNA‐CN), and leukocyte telomere length. This study shows that treatment with plasma concentrate is safe. More than 20 clinical biomarkers were significantly and beneficially altered following the treatments. For example, creatinine was significantly decreased (*p* = 0.0039), while estimated glomerular filtration rate (eGFR) was increased (*p* = 0.0044), indicating the treatment may improve biomarkers of kidney function. Three of four immunoglobulin biomarkers decreased, while telomere length and mtDNA‐CN were not significantly affected by the treatment. The treatment reduced DNA methylation‐based GrimAge by an average of 0.82 years (*p* = 0.0093), suggests a reduction in morbidity and mortality risk. By contrast, no significant results could be observed for epigenetic clocks that estimate chronological age. Our results support the view that plasma concentrate contains youth‐promoting factors.

## Graphical abstract

We conducted a safety study (*n* = 18, mean age 74) by injecting human umbilical cord plasma concentrate (hereinafter Plasma Concentrate) weekly (1 ml intramuscular) into elderly human subjects over a 10‐week period. Our results indicate that treatment with plasma concentrate is safe. In addition, the treatment reduced DNA methylation‐based GrimAge, suggesting a reduction in morbidity and mortality risk. Taken together, our results support the view that plasma concentrate contains youth‐promoting factors.

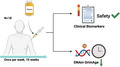

## 1 INTRODUCTION

Inflammaging (or inflamm‐aging) is a concept that describes the chronic inflammation that occurs with the progression of aging even in the absence of overt infection. It is not only a hallmark of aging (Lopez‐Otin et al., 2013), but also considered a potential cause of senescence and aging (Ferrucci & Fabbri, 2018). Although there is no treatment that reverses the detrimental effects of aging yet, many laboratories, including ours, have started to study interventions that would lower such systemic sterile inflammation to potentially slow down the progressive decline of health and homeostatic recovery that are associated with advanced aging. Out of many interventions, here we evaluated the effect of a variation upon therapeutic plasma exchange (TPE), which consists of providing molecular components of donor blood plasma as a therapeutic agent to a patient.

TPE, also known as therapeutic apheresis or simply plasma exchange, is not a new therapeutic modality. This is evidenced by the 2011 National Blood Collection and Utilization Survey Report, which notes that the amount of fresh frozen plasma transfused into patients in the United States annually had reached over 4.5 million units (Ellingson et al., 2017, p.2015). Plasma transfusions are now considered a standard treatment for many different indications, including inflammatory or autoimmune conditions (especially antibody‐mediated disorders), rare genetic diseases related to blood clotting, sepsis and critical illness, familial hypercholesterolemia, where low‐density lipoprotein (LDL) and large molecules related to premature atherosclerotic cardiovascular disease can be removed, among others (Padmanabhan et al., 2019). Therapeutic apheresis has also been studied in tens of thousands of patients in diverse settings, with adverse reactions occurring only rarely (in approximately 4% of procedures) (Sha et al., 2019).

Plasma is a straw‐colored liquid that contains multiple components, including minerals, vitamins, RNA, DNA, and proteins such as albumin, coagulation factors, fibrinogen, various globulins including immunoglobulins, complement components, cytokines, and hormones. While blood plasma does not contain blood cells or platelets, it does contain microvesicles such as exosomes. Exosomes are between 30 and 150 nanometers in size, and are involved in specialized functions, including waste management and signaling between cells (van der Pol et al., 2012). Exosomes may be the vehicles by which stem cells release their paracrine factors to other cells and thereby exert their regenerative effects in many tissues (Han et al., 2016). This is exemplified by the fact that the exosomes of mesenchymal cells have been shown to aid in wound healing through the use of growth factors (hepatocyte growth factor (HGF), insulin‐like growth factor (IGF1), nerve growth factor (NGF), and stromal‐derived growth factor (SDF1)). Additionally, human fibrocyte‐derived exosomes have been shown to have a role in wound healing in diabetic mice through an anti‐inflammatory mechanism involving heat shock protein‐90α and micro RNAs (Geiger et al., 2015).

Although we do not fully understand all the plasma components, we know that not all plasma is the same and that the quality, quantities, and ratios between molecules change as we age. In addition, we know that plasma exchange via heterochronic parabiosis where the circulatory systems of young and old rodents are conjoined, beneficially affects the health of the old animals while detrimentally affecting the young animals (Conboy et al., 2005). In 2014, pioneering researchers began studying plasma infusion treatment for therapeutic uses, including for reducing inflammation in Alzheimer's disease (Villeda et al., 2014). They described injecting middle‐aged mice with plasma from young mice, which more closely simulates how treatment could be delivered in a clinical setting (Villeda et al., 2014). After three weeks, the mice displayed improvements in synaptic plasticity and certain measures of cognitive function. There was even some improvement in mouse models of familial Alzheimer's disease (Middeldorp et al., 2016). A similar experiment in mice was later repeated using human umbilical cord plasma, and similar results were noted, including the revitalized function of the hippocampus (Castellano et al., 2017). Multiple umbilical cord blood plasma infusions have been given to animals and, even in immunodeficient animal models, did not cause detrimental immune responses (Castellano et al., 2017).

Therapy with mesenchymal stem cells (MSCs) has been proposed as one option to treat the detrimental health effect of aging (i.e., frailty), even though the mechanism of action of MSCs is not yet well established. Studies in rats suggest that human placenta‐derived MSCs increase multiple factors associated with a prolonged, healthy life span, such as vitamin B6, and proteins associated with hepatocyte proliferation and mitochondrial biogenesis in aged female rats (Kim & Lee, 2022). MSCs have long been recognized for their immuno‐modulatory (Rasmusson et al., 2005) and growth factor promoting properties (Ranganath et al., 2012), primarily exerted via paracrine and other secretory processes (Gnecchi et al., 2008). This suggests the possibility of using secretory molecules of MSCs as the therapeutic agent without needing a transfusion of living cells. Early‐stage studies using human umbilical cord MSCs have shown promising results in treating frailty (Florea et al., 2019), and the human umbilical cord represents not only an ethical and easily obtained source of therapeutic cells, but umbilical cord blood and plasma can also be used as a source from which to collect and concentrate their secreted molecules (the “secretome”) (Nagamura‐Inoue & He, 2014).

Thus, in addition to the use of umbilical cord MSC cells, umbilical cord plasma has been considered as a source of young secretome factors that could aid in the reversal or “treatment” of aging. For example, in October 2014, a startup (Alkahest) began recruiting participants for a trial at Stanford School of Medicine, using young blood in people with late‐stage Alzheimer's disease (https://clinicaltrials.gov/ct2/show/NCT02256306). The trial was completed in February of 2017. This was a phase I trial that established safety and the company is currently conducting a phase II trial with human Umbilical Cord Blood Plasma (hUCBP). In 2015, Bundang CHA General Hospital in South Korea launched a clinical trial to compare the anti‐aging effects of umbilical cord blood, young plasma, and placebo on markers of frailty in aging (See https://clinicaltrials.gov/ct2/show/NCT02418013).

In this study, we sought to use plasma‐derived from human umbilical cord blood as a therapeutic source, as it was not only—“younger” (relative to subjects' ages), but enriched with immuno‐modulating factors and growth factors. This is the first case that we know of involving intramuscular injections of a concentrate of plasma obtained by centrifugation rather than by supplying the plasma as an infusion. The purpose of this IRB‐approved safety study was to determine whether multiple injections of the plasma concentrate (each injection derived from 100 ml of umbilical cord plasma) given on a weekly basis for a total of ten weeks (the total concentrate thus the equivalent of receiving 1 liter of plasma secretory factors) would be well tolerated in elderly patients, and what effects such treatment would have on the overall inflammation levels and general health of the participants.

For a preliminary evaluation of treatment efficacy, we focused on established clinical biomarkers and DNA methylation‐based age and mortality risk estimators known as epigenetic clocks (reviewed in [Horvath & Raj, 2018]). Large retrospective epidemiological studies have linked GrimAge to mortality risk, incident functional decline, and the onset of several age‐related diseases, including heart disease, cancer onset, multi‐modal measures of brain health, kidney disease, fatty liver, and diminished respiratory function (Lu, Quach, et al., 2019; McCrory et al., 2020). GrimAge has been used in several previous intervention trials as a blood‐based indicator of biological age (Fahy et al., 2019; Li et al., 2020)

## 2 RESULTS

### 2.1 Enrolled study population

This study investigated primarily the safety of treatment in 18 participants (10 women and 8 men), who were recruited after meeting the study's inclusion and exclusion criteria (Methods). The age of the participants ranged from 60 to 95, with an average age of 74 (Table S1). Women were slightly younger than men (mean age 71 vs 78). The average weight of men and women was 197 pounds and 146 pounds, respectively (Table S1).

### 2.2 Clinical biomarkers

In total, we measured 78 clinical biomarkers for each participant before and after the treatment. Pearson correlations between biomarkers can be found in Figure S1. We conducted paired t‐tests to investigate whether clinical biomarkers were changed after the treatment. To adjust for multiple comparisons, we used a false discovery rate (FDR) approach (Benjamini–Hochberg implemented in the R function p.adjust).

**FIGURE 1:**
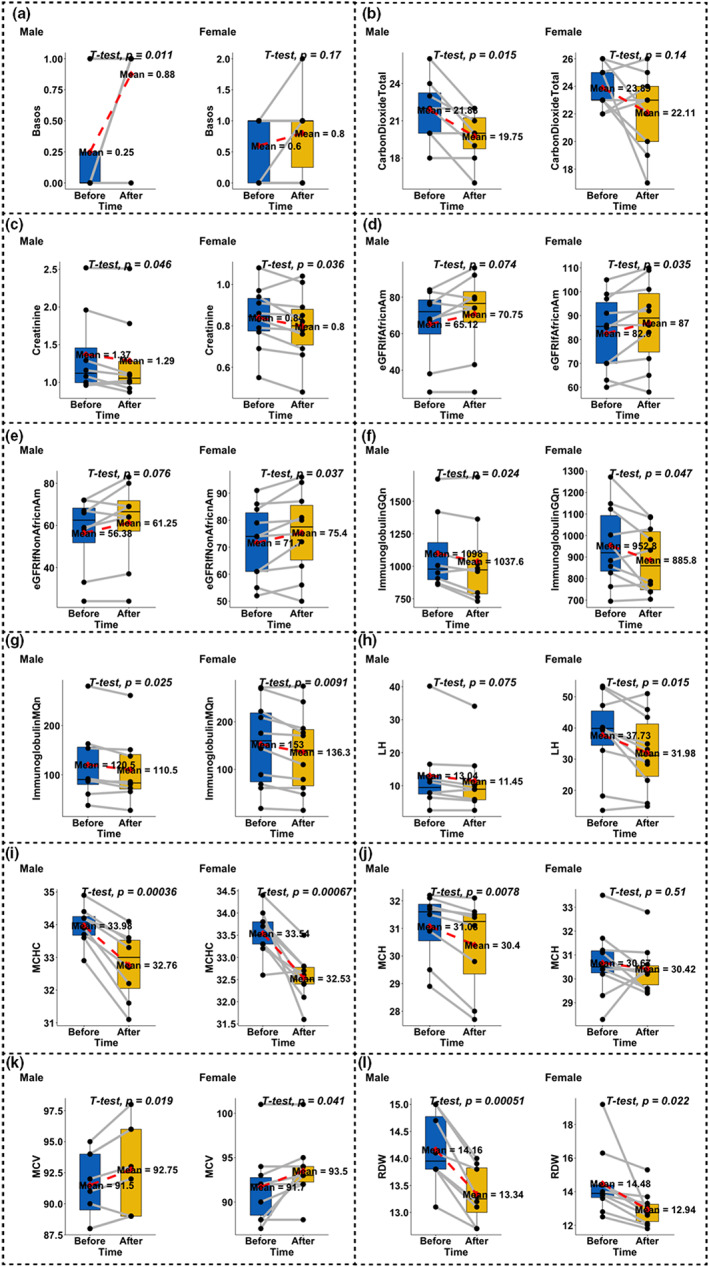
Evaluation of clinical biomarkers. We only present results for biomarkers that led to an unadjusted significance level of *p* < 0.01. The x‐axis represents before and after the treatment. Two dots connected by gray lines represent the same participant. *p*‐values were derived from paired t‐tests. Males and females were tested separately. (a) Basophils, (b) carbon dioxide (total amount), (c) creatinine, (d) estimated glomerular filtration rate (African American), (e) estimated glomerular filtration rate (non‐African American), (f) immunoglobulin G, quantitative, (g) immunoglobulin M, quantitative, (h) luteinizing hormone, (i) mean corpuscular hemoglobin concentration, (j) mean corpuscular hemoglobin, (k) mean corpuscular volume, and (l) red blood cell distribution width

In total, 10 out of 78 biomarkers were significant at FDR <0.05 (Figure 1, Table S2). Red blood cell indices, including the red blood cell distribution width (RDW, nominal *p* = 0.0014) and mean corpuscular hemoglobin concentration (MCHC, *p* = 3.5E‐7), decreased significantly after the treatment for both males and females, while mean corpuscular volume (MCV, *p* = 3.1E‐3) increased significantly (Figure 1, Tables S2–S3). Among all four immunoglobulins (IgA, E, G, and M), two (G and M) did go down significantly over the treatment period for both sexes. Two indicators of kidney function, that is, creatinine and estimated glomerular filtration rate (eGFR), were altered after the treatment. Specifically, creatinine level decreased after the treatment (*p* = 3.9E‐3) and eGFR values increased (*p* = 4.4E‐3). Among various hormones tested, the level of luteinizing hormone (LH) declined after the treatment for both males and females. During the treatment, blood carbon dioxide decreased in both men and women, but the effect was more pronounced in men. In addition, basophils (Basos) were elevated in both sexes, with a greater effect in males (Figure 1, Table S3).

### 2.3 Safety and tolerability

Since repeated injections of a substance containing proteins can trigger an unwanted immune response, we assessed clinical symptoms of immune reactions and measured parameters to assess humoral and cellular immune reactions. We found that immunoglobulins A, G, and M were actually significantly decreased, rather than increased. The level of immunoglobulin E was not significantly changed. With regard to cellular immunity, we found no significant change in the level of whole blood cells, neutrophils, monocytes, or plasma blasts. We found no significant change in lymphocyte numbers and no change in naive CD8+ or CD4+ T cells. However, we found significant increases in eosinophils and basophils (*p* < 0.05) after treatment. Two female patients with autoimmune issues had redness and heat at the site of the needle puncture within a few hours of receiving their first injection and were treated with an injection of diphenhydramine (Benadryl) without further distress. For the next two weeks, they both received a pre‐treatment injection of diphenhydramine, with no reaction resulting from the plasma concentrate injection, and by the fourth week, they were taking the injection without indications of any adverse reactions. No other significant adverse effects in other patients were observed or reported.

### 2.4 Epigenetic clock analysis

Peripheral whole blood samples were collected for each participant, and DNA methylation was measured using the HumanMethylationEPIC Bead Chip (Illumina, San Diego, CA). We then calculated several human epigenetic biomarkers of aging (epigenetic clocks) and estimated cell compositions based on blood methylation data: (1) The pan‐tissue epigenetic age (referred to as DNAmHorvath) (Horvath, 2013); (2) Hannum's blood‐based DNAm age (DNAmHannum) (Hannum et al., 2013); (3) skin and blood clock (DNAmAgeSkinClock) (Horvath et al., 2018); (4) DNAm of the surrogate markers of telomere length (DNAmTL) (Lu, Seeboth, et al., 2019); (5) DNAmPhenoAge (Levine et al., 2018); (6) the mortality risk estimator DNAmGrimAge and its components (Lu, Quach, et al., 2019); and (7) estimated cell compositions including naive CD8 cells (CD8.naive), exhausted CD8+ cells (CD8pCD28nCD45Ran), plasma blasts, CD4 T cells (CD4T), natural killer cells (NK), monocytes (Mono), and granulocytes (Gran). To formally measure possible epigenetic age acceleration (AgeAccel) effects, we fit a regression model of DNAm age on chronological age and defined AgeAccel as the resulting raw residual. Thus, positive AgeAccel means the methylation state of the sample appears to be older than would be expected based on chronological age. Before and after samples for all participants were retained, and one outlier sample was retested.

**FIGURE 2:**
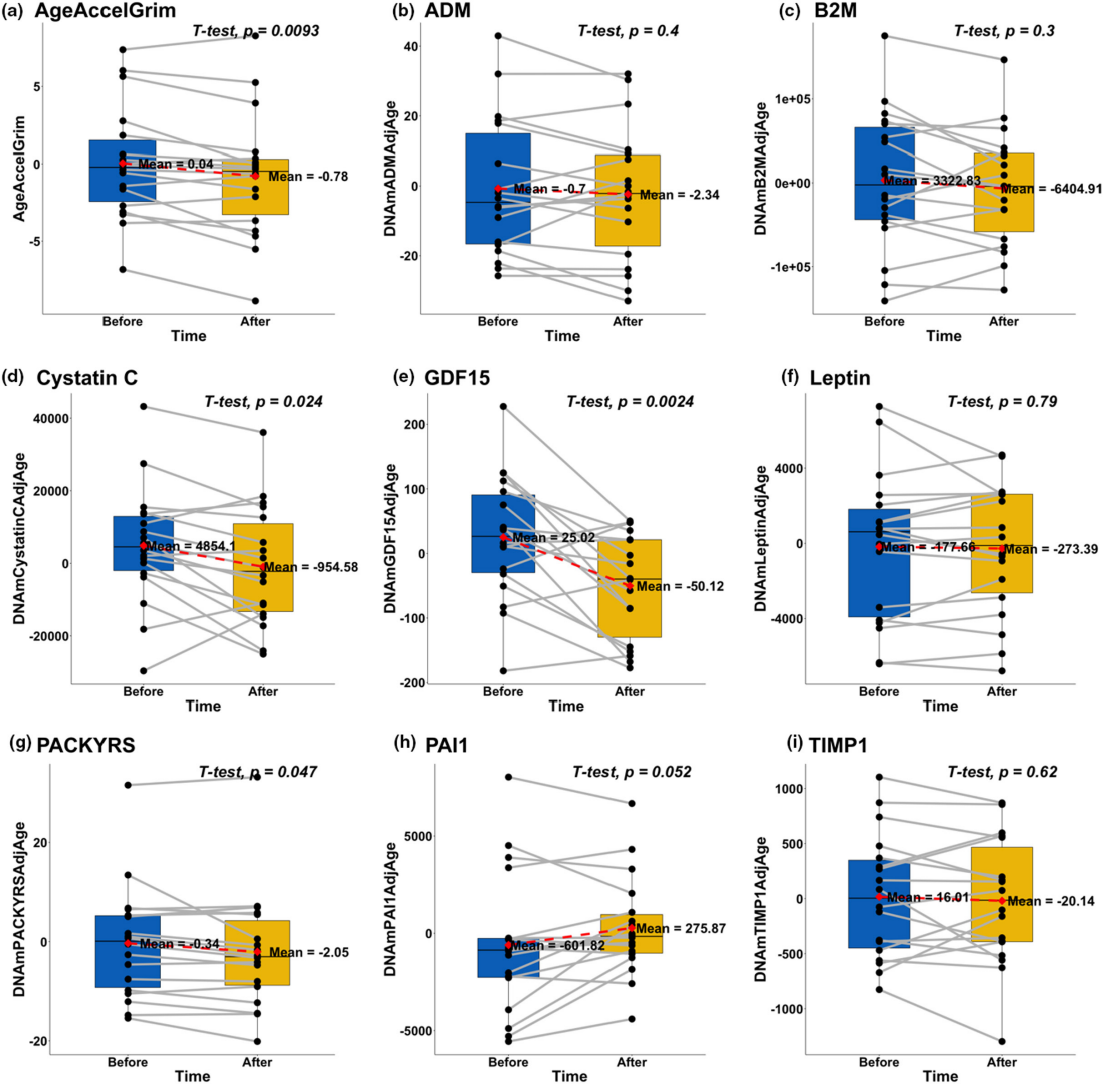
Treatment effect on GrimAge age acceleration (AgeAccelGrim) and its components. (a) Grim AgeAccel is defined as the raw residuals derived from regression models of DNAmGrimAge on chronological age. (b–i) 8 GrimAge components: DNAm‐based estimators of (b) adrenomedullin (ADM), (c) beta‐2‐microglobulin (B2M), (d) cystatin C (cystatin C), (e) growth differentiation factor‐15 (GDF15), (f) leptin, (g) smoking pack‐years (PACKYRS), (h) PAI‐1 (PAI1), and (i) tissue inhibitor metalloproteinases 1 (TIMP1), respectively. Two‐sided uncorrected *p*‐values were derived from paired student's t‐tests

Pairwise correlations between epigenetic biomarkers, blood cell compositions, and chronological age are presented in Figure S2. AgeAccelGrim was significantly decreased from 0.04 to −0.78 (paired Student's t‐test *p* = 9.3E‐3) (Figure 2, Table S2). The other epigenetic clocks were not significantly changed following the treatment (Figure S3).

We also tested whether components of DNAmGrimAge were altered in response to the treatment since DNAmGrimAge can be interpreted as a linear combination of DNAm‐based biomarkers for smoking pack‐years (PACKYRS) and 7 DNAm‐based estimators of plasma protein biomarkers, including adrenomedullin (ADM), beta‐2‐microglobulin (B2M), cystatin C, growth differentiation factor‐15 (GDF15), leptin, plasminogen activator inhibitor 1 (PAI1), and tissue inhibitor metalloproteinases 1 (TIMP1). Based on paired Student's t‐test and after adjusting for age, we observed that the treatment significantly lowered DNAm Cystatin C (*p* = 2.4E‐2) and DNAm GDF‐15 (*p* = 2.4E‐3).

We also investigated whether DNAm‐based estimation of cell compositions was associated with the treatment. Out of 7 cell types, only monocytes were significantly increased after the treatment (nominal *p* = 1.6E‐2, Figure S4, Table S2), but the finding is not significant after adjusting for multiple (seven) comparisons.

### 2.5 Stratification by sex

For the sake of completeness, we also present the results of a sex‐stratified analysis even though our study is not properly powered to carry such an analysis (*n* = 10 women and *n* = 8 men).

DNAmGrimAge significantly decreased in women (*p* = 2.2E‐2, Table S4) but not in men (*p* > 0.2). Several component traits of DNAmGrimAge were significantly lowered in women: age‐adjusted DNAm estimates of GDF15 (*p* = 1.6E‐2), cystatin C (*p* = 8.8E‐3), and the methylation‐based estimate of smoking packyears (*p* = 0.042) but not in men, while PAI1 was significantly increased in women (*p* = 1.5E‐2) (Table S4).

DNAm age acceleration, according to the pan tissue clock, showed a suggestive decrease in men (*p* = 5.4E‐2) but an increase in women (*p* = 1.7E‐2). We believe these opposite directions could be false‐positive associations due to low sample sizes.

### 2.6 Stratification by age

In order to investigate the treatment effect across different age groups, we stratified the participants based on their chronological age at enrollment (*n* = 9 younger than or equal to 75, and *n* = 9 older than 75).

Overall, the treatment affected the clinical biomarkers in the same direction between different age groups. Among the 10 biomarkers significantly affected by the treatment in the primary analysis, Basos, IgG, MCHC, and MCV showed more significant responses among participant older than 75, and the effects on Creatinine, eGFR, IgM, LH, and RDW were more pronounced in participants younger than 75 (Table S5).

In addition, the treatment had stronger effect of lowering age‐adjusted DNAm estimates of GDF15 in participants younger than 75 (*p* = 2.6E‐2), while effect on Cystatin C was more significant in participants older than 75 (*p* = 2.2E‐2, Table S6).

### 2.7 Telomere length and mitochondrial copy number

Leukocyte telomere length was not significantly associated with the treatment. However, we find suggestive evidence (*p* = 7.5E‐2, Figure S5) that the post‐treatment mtDNA levels were increased.

### 2.8 Treatment increased mean methylation and decreased Shannon entropy

The treatment slightly increased the mean methylation levels (averaged across the 866,238 CpGs on the array) based on paired‐wise t‐test (difference = 0.003, *p* = 5.0E‐3). In addition, treatment significantly increased mean methylation levels of CpG probes within 5′ untranslated regions (5'UTR), gene body, and 3' UTR (Table S7).

We calculated the Shannon entropy of the whole methylome based on the EPIC array. An increase in entropy means that the methylome becomes noisier, that is, when the methylation value (beta values) regress toward 50%. Specifically, we calculated the entropy as follows

$$\text{Entropy}=\frac{1}{N\times \log_{2}\left(\frac{1}{2}\right)}\times \sum_{i=1}^{N}\left[\beta_{i}\times \log_{2}\left(\beta_{i}\right)+\left(1-\beta_{i}\right)\times \log_{2}\left(1-\beta_{i}\right)\right]$$

where $\beta_{i}$ represents the methylation beta value for the *i* ^th^ probe (CpG site) in the array, N represents the total number of CpGs included in the formula.

**FIGURE 3:**
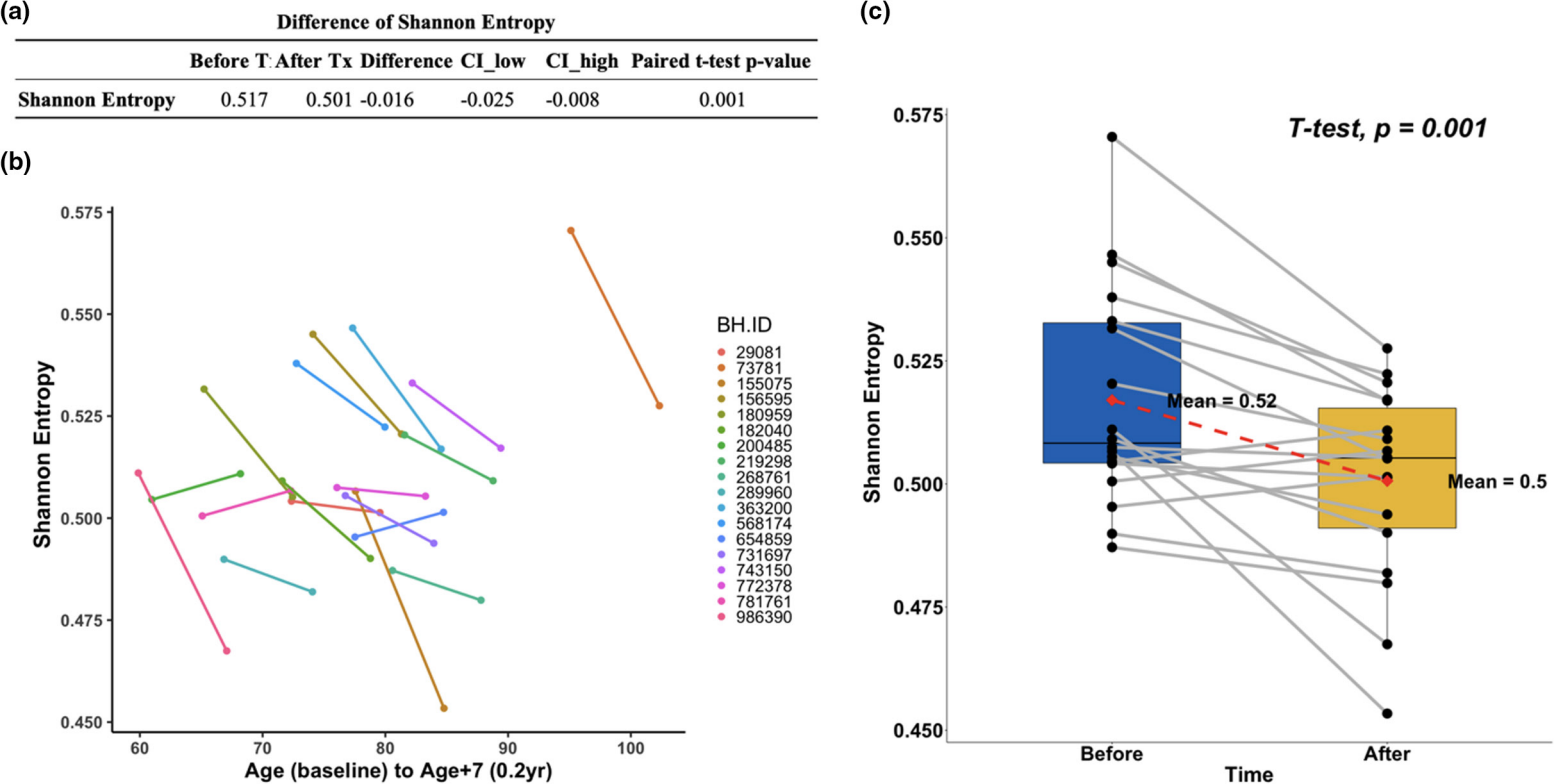
Changes in methylation entropy. (a) Results of the paired t‐test. (b) Spaghetti plots. Age after treatment is represented by artificially calculating 7 additional years for better visualization. (c) Paired boxplot. The y‐axis shows the Shannon entropy level. Uncorrected two‐sided *p*‐value was calculated with a paired Student's *t*‐test.

We found that the entropy was significantly decreased after the treatment (*p* = 1.0E‐3) (Figure 3).

### 2.9 Epigenome‐wide association analysis

**FIGURE 4:**
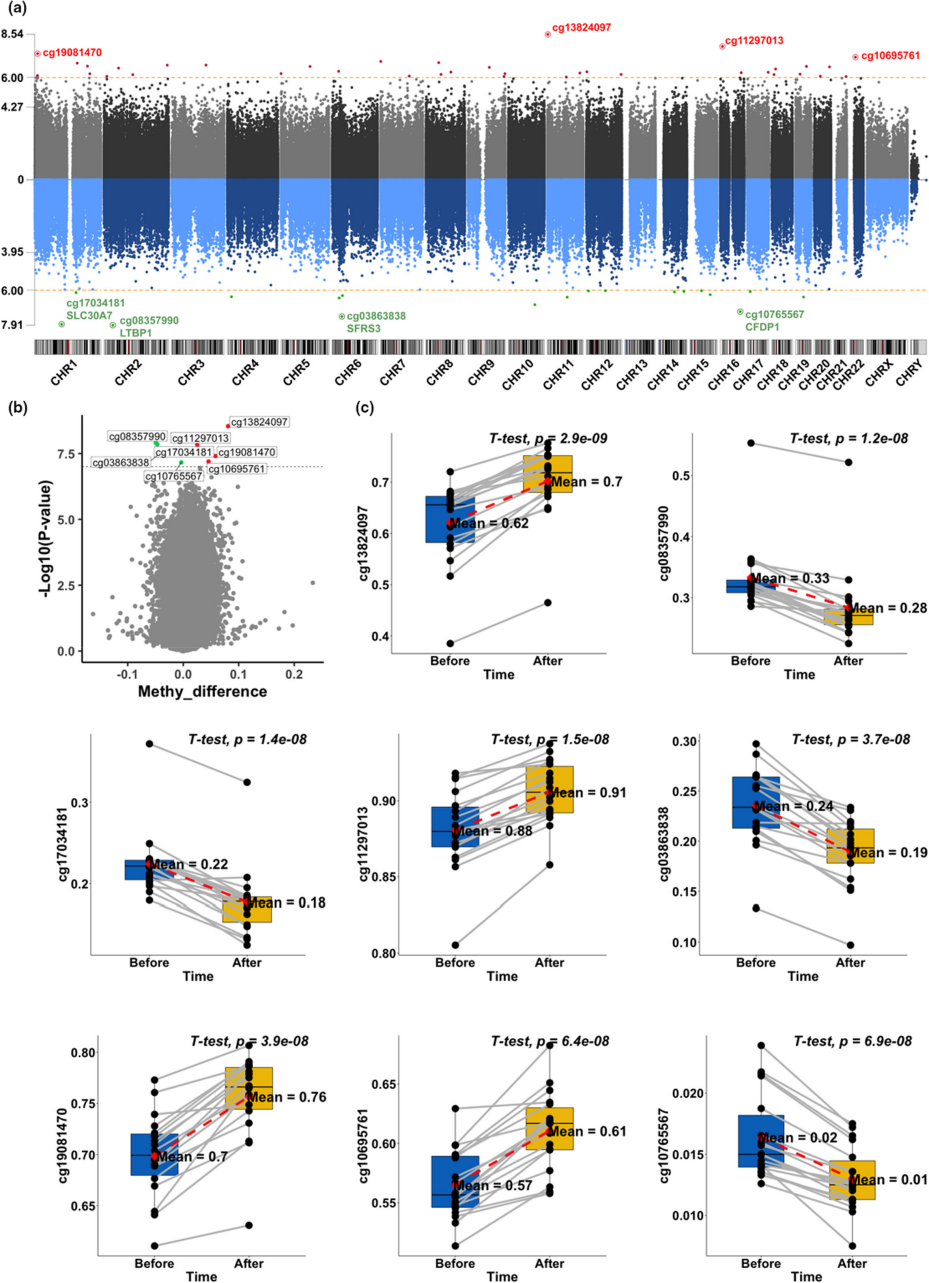
EWAS of treatment effects. Manhattan plot (a) shows the significance of the associations between individual cytosine methylation levels and the treatment. The y‐axis shows the –log_10_ transformed *p*‐value from a paired student's t‐test. Red and green dots represent CpGs that gained or lost methylation with the treatment, respectively. Volcano plot (b) shows the *p*‐values and changes in methylation level in response to the treatment. The y‐axis shows the ‐log10(p). The x‐axis shows the mean difference in methylation level before and after the treatment for each CpG. In addition, paired boxplots (c) of top CpGs with raw *p* < 1E‐7. The y‐axis shows the methylation level.

We conducted paired t‐test to identify CpGs related to the treatment. QQ‐plot and distribution of raw *p*‐values can be found in Figure S6. Strong inflation was observed based on the inflation factor (λ = 3.49). In total, 8 CpGs were significant at an unadjusted *p* < 1E‐7 level, and an additional 47 CpGs were significant at an unadjusted *p* = 1E‐6 level, mapping to 34 genes (Table 1, Figure 4a–c). Among the 55 CpGs with *p* < 1E‐6, 37 were hypermethylated (67.3% vs. 32.7%).

**TABLE 1:** Top 20 DNA methylation sites significantly associated with the treatment

Chr	Probe	Bp	CpG island	Gene	Gene loc	Difference	CI_low	CI_high	*p*‐value	FDR
11	cg13824097	1,827,023				0.081	0.096	0.066	2.89E‐09	2.50E‐3
2	cg08357990	33,359,550		LTBP1	TSS200	−0.049	−0.039	−0.060	1.24E‐8	3.23E‐3
1	cg17034181	101,362,442	S_Shore	SLC30A7	Body	−0.047	−0.037	−0.056	1.41E‐8	3.23E‐3
16	cg11297013	8,346,717				0.025	0.031	0.020	1.49E‐8	3.23E‐3
6	cg03863838	36,562,081	Island	SFRS3	TSS200	−0.046	−0.036	−0.056	3.70E‐8	5.67E‐3
1	cg19081470	11,000,722				0.058	0.071	0.045	3.93E‐8	5.67E‐3
22	cg10695761	21,539,377	S_Shore			0.046	0.056	0.035	6.36E‐8	7.44E‐3
16	cg10765567	75,467,484	Island	CFDP1	TSS200	−0.003	−0.003	−0.004	6.87E‐8	7.44E‐3
7	cg05725489	600,658	N_Shore	PRKAR1B	Body	0.030	0.037	0.023	1.12E‐07	1.08E‐2
8	cg23628099	48,089,762				0.051	0.063	0.038	1.33E‐07	1.11E‐2
1	cg07907639	159,787,620				0.031	0.039	0.023	1.41E‐07	1.11E‐2
10	cg07834955	99,531,879	Island	SFRP5	TSS200	−0.006	−0.005	−0.008	1.63E‐07	1.13E‐2
3	cg26946032	129,305,723		PLXND1	Body	0.017	0.022	0.013	1.84E‐07	1.13E‐2
2	cg11865801	237,967,595				0.016	0.020	0.012	1.86E‐07	1.13E‐2
1	cg24710995	198,431,936				0.031	0.039	0.023	2.14E‐07	1.13E‐2
19	cg00772380	41,135,151	N_Shore	LTBP4	Body	0.025	0.031	0.019	2.19E‐07	1.13E‐2
5	cg00398767	109,037,023		MAN2A1	Body	0.034	0.043	0.026	2.22E‐07	1.13E‐2
20	cg22659862	57,438,238		GNAS	Body	0.012	0.014	0.009	2.36E‐07	1.13E‐2
9	cg09870092	80,938,588		PSAT1	Body	0.051	0.064	0.038	2.51E‐07	1.14E‐2
2	cg22708752	54,853,191	N_Shelf	SPTBN1	Body	0.005	0.007	0.004	2.78E‐07	1.20E‐2

The most significant CpG site is cg13824097 (intergenic, chr11:1,827,023), for which methylation level was increased after the treatment (*p* = 2.9E‐09). Other top hits include cg08357990 in *LTBP1* (*p* = 1.2E‐8), cg17034181 in *SLC30A7* (*p* = 1.4E‐8), cg03863838 in *SFRS3* (*p* = 3.7E‐8), and cg10765567 in *CFDP1* (*p* = 6.9E‐8), which were all decreased after the treatment (Table 1). Moreover, methylation levels of several intergenic CpGs were significantly increased after the treatment, including cg11297013 (intergenic, chr16: 8,346,717), cg19081470 (intergenic, chr1: 11,000,722), and cg10695761 (intergenic, chr22: 21,539,377).

**FIGURE 5:**
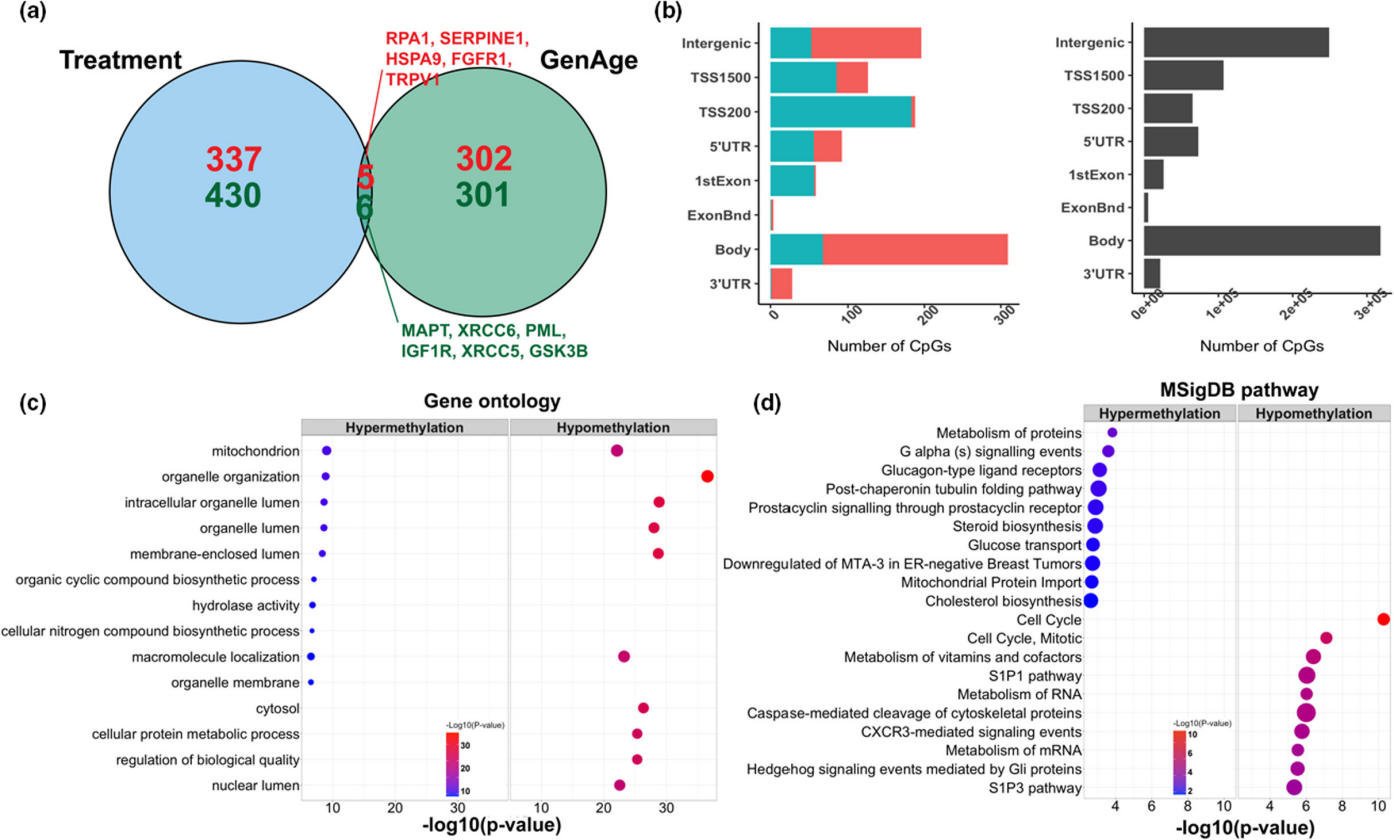
Functional enrichment analyses of treatment‐related CpGs. Venn diagram (a) shows the overlap between genes within the GenAge database and genes annotated to the top 500 hyper /hypomethylated CpGs in our study. (b) Location of the top CpGs relative to the closest transcriptional start site. The gray color in the right panel represents the location of all EPIC array probes mapped to the human hg38 genome. (c–d) Gene ontology and MSigDB pathway enrichment analysis based on GREAT. The left panel shows the top enriched GO terms based on the top 500 hyper/hypomethylated CpGs. In addition, the right panel shows the top enriched MSigDB pathways. The size of the dot represents the enrichment ratio. GO terms were simplified for easier interpretation by calculating the similarity of GO terms and removing those highly similar terms using rrvgo.

To investigate the relationship between top CpGs associated with the treatment and known aging‐related genes, we compared our results with the GenAge database (Tacutu et al., 2018), a curated database of over 300 genes related to human aging. Among genes annotated to the top 500 CpGs that gain methylation and top 500 CpGs that lose methylation in response to the treatment (raw *p* < 5E‐5), 11 were known aging‐related genes according to the GenAge database (Figure 5a). These genes include *RPA1* (*p* = 1.2E‐06), *SERPINE1* (*p* = 2.2E‐06), *HSPA9* (*p* = 9.0E‐06), *FGFR1* (*p* = 1.8E‐05), *TRPV* (*p* = 2.0E‐05), *MAPT* (*p* = 5.6E‐06), *XRCC6* (*p* = 6.5E‐06), *PML* (*p* = 1.3E‐05), *IGF1R* (*p* = 1.4E‐05), *XRCC5* (*p* = 2.0E‐05), and *GSK3B* (*p* = 3.2E‐05).

Because monocytes were significantly increased after the treatment, which could potentially bias the EWAS results, we conducted a sensitivity analysis by adjusting for monocytes in the model. In the sensitivity analysis, we identified 25 CpGs with an unadjusted *p* < 1E‐6, 18 of which were also significant in the original results with an unadjusted *p* < 1E‐6 (Table S8).

### 2.10 Functional enrichment analysis

To uncover biological processes potentially linked to treatment‐related CpGs, we employed a series of functional enrichment analyses to characterize treatment‐related CpGs (Figure 5b–d). For the sake of statistical power, we lowered the significance threshold so that we would end up with 500 CpGs that gain methylation in response to the treatment and 500 CpGs that lose methylation.

The top 500 CpGs that gained methylation following the treatment were significantly enriched in CpG island shelves (11% of the 500 CpGs are in shelves, whereas 7% of all CpGs are in shelves, hypergeometric *p* = 6.0E‐4) and open sea area (*p* = 2.4E‐5). The top 500 CpGs that lost methylation following the treatment were significantly enriched in CpG islands (75% vs. 18%, *p* = 6.0E‐166) and shores (*p* = 3.0E‐3). In addition, hypomethylated CpGs were also significantly enriched in transcriptional start site TSS200 (36.4% vs. 7.5%, *p* = 5.4E‐76), 5'UTR (10.8% vs. 8.4%, *p* = 3.0E‐2), and 1stExon (11.0% vs. 3.1%, *p* = 1.5E‐16) (Figure 5b). These results indicated that CpGs in transcription regulatory regions (promoters, 5'UTRs, and CpG islands) have negative associations with the treatment (Figure 4b). Interestingly, in contrast, a previous study showed that age‐related CpGs have methylation increasing with age within these regulatory regions.

Noteworthy genes adjacent to top EWAS CpGs include *CFDP1* (Cranio Facial Development Protein 1) and *PRKAR1B* (Protein Kinase CAMP‐Dependent Type I Regulatory Subunit Beta) which are related to fibroblastic disorders, while *MAN2A1* (Mannosidase Alpha Class 2A Member 1), *PSAT1* (Phosphoserine Aminotransferase 1), *GNAS* (GNAS Complex Locus), and *SPTBN1* (Spectrin Beta, Non‐Erythrocytic 1) are linked with cystic fibrosis (Chen et al., 2013).

We also employed the Genomic Regions Enrichment of Annotations Tool (GREAT) to identify biological functions associated with genes annotated to treatment‐related CpGs. The enrichment analyses of the genes adjacent to the top 500 positively and the top 500 negatively treatment‐related CpGs identified overrepresentation of biological processes involving mitochondrial function, organelle organization and macromolecule localization, and cellular components including membrane‐enclosed lumen and cytosol (Figure 5c, Table S9). MSigDB pathway enrichment analysis identified top enriched pathways, including cell cycle‐related pathways, metabolism of vitamins and cofactors, sphingosine 1‐phosphate (SIP) related pathways, and metabolism of RNA (Figure 5d, Table S10). The rest of functional enrichment results can be found in Figure S7.

## 3 DISCUSSION

This safety study demonstrated that plasma concentrate treatment (intramuscular injections of 1 ml) was well tolerated. A large body of literature has shown that young blood plasma has beneficial effects on murine organs (Castellano et al., 2017; Middeldorp et al., 2016). Our study is the first human study to investigate whether umbilical cord‐plasma concentrate reverses human epigenetic age according to widely used epigenetic clocks. We find a nominally significant age‐reversal effect for DNAmGrimAge, which is the most robust epigenetic predictor of morbidity and mortality risk in humans. We detect significant reversal of the DNAmGrimAge after the plasma concentrate treatment (from 0.04 to −0.78).

DNAmGrimAge can be interpreted as a linear combination of seven DNAm‐based estimators of plasma proteins and DNAm estimates of smoking pack years. Secondary analyses revealed that two of these underlying epigenetic biomarkers (estimated Cystatin C levels and growth differentiation factor 15, GDF‐15) were significantly decreased after the plasma concentrate treatments. Consistent with these findings for epigenetic biomarkers, we also observe that clinical cystatin C measurements were reduced following the treatment.

Cystatin C is a cysteine proteinase inhibitor that is produced by nearly all human cells and released into the circulation. Plasma cystatin C is used to assess kidney function (Ferguson et al., 2015), and a higher level of cystatin C has been associated with increased risk of many age‐related traits, such as chronic obstructive pulmonary disease (COPD) and cardiovascular disease (CVD) (Lu, Quach, et al., 2019).

GDF15 belongs to the transforming growth factor‐β (TGF‐β) superfamily of proteins. Levels of GDF15 in the blood are elevated with age, as well as in response to cellular stress and mitochondrial malfunction (Rochette et al., 2021). Previous epidemiological studies showed that increased GDF15 was significantly associated with cardiovascular and all‐cause mortality (Daniels et al., 2011).

Consistent with the finding that DNAm‐based Cystatin C estimator increased after the treatment, we also observed two clinical biomarkers of kidney function, creatinine and estimated glomerular filtration rate (eGFR) were altered after the treatment (creatinine levels decreased after the plasma concentrate treatment and eGFR values increased). One study with 8913 healthy participants indicated lower GFR and mild renal failure could be a risk factor for cardiovascular and all‐cause mortality (Van Biesen et al., 2007).

The plasma concentrate treatment also had beneficial effects on several other established clinical biomarkers. We observed that red blood cell distribution width (RDW) decreased significantly after the treatment. RDW is a measure of heterogeneity in the size of circulating erythrocytes, and it has been proposed as a potential screening marker for various diseases such as stroke, celiac disease, and cardiovascular and cerebrovascular diseases (Li et al., 2017).

In order to gain more insights into the mechanism of the plasma concentrate treatment, we investigated the changes in DNA methylation at the whole genome level. From our EWAS results, we identified two latent transforming growth factor‐β binding protein genes (LTBP), *LTBP1* and *LTBP4*, as genes that are proximal to cytosines whose methylation changes significantly with the treatment. LTBPs are components of the extracellular matrix (ECM) and local regulators of transforming growth factor‐β (TGF‐β) tissue deposition and signaling (Sterner‐Kock et al., 2002). Interestingly, we also observed several other top treatment‐related genes associated with fibroblast disorders, fibrosis the extracellular matrix (ECM). Taken together, our results suggested a potential role of plasma concentrate treatment for ECM homeostasis, which plays a role in healthy aging (Freitas‐Rodriguez et al., 2017). In addition, functional enrichment studies revealed that the treatment affected CpGs that are located near nuclear mitochondrial genes and genes that play a role in mitochondrial protein import. Mitochondrial function is known to be disrupted during the aging process (Lopez‐Otin et al., 2013). Consistently, a direct measure of mitochondrial copy number in blood revealed a nominally significant increase.

The current study has several strengths. First, this study involved humans as opposed to rodents. Second, deep phenotyping was employed, including clinical biomarkers, leukocyte telomere length, and mitochondrial copy number. Third, we conducted detailed investigations on epigenetic clocks and DNA methylation across the genome. There are also several limitations. First, this was not a placebo‐controlled randomized clinical trial. This safety study was intended to primarily establish safety as opposed to efficacy of the treatments. Second, the treatment only affected a single epigenetic clock (DNAmGrimAge) which indicates that the treatment effect could be relatively weak. Other epigenetic clocks, notably first‐generation epigenetic clocks that measure chronological age, failed to detect a significant age‐reversal effect.

We demonstrate that the treatment decreased the entropy in the methylation data. Due to the failure of DNAm maintenance, the Shannon entropy measurement increases with age, indicating a less predictable methylome across a population of cells (Hannum et al., 2013). In addition, a previous study has shown a significant correlation between DNAm‐based age acceleration and entropy (Yan et al., 2020). Here, the decreased entropy may implicate the rejuvenation of the epigenetic landscape after the plasma concentrate treatments. Recent experiments with plasma exchange apheresis raised the possibility that the benefit of treatment with young plasma is primarily due to the dilution of old plasma and a molecular resetting of the systemic signaling milieu (Mehdipour et al., 2020). Mehdipour et al. (2020) observed molecular resetting of the systemic signaling milieu by replacing half of the plasma in mice with saline containing 5% albumin. Tang et al. (2021) reported that young and undamaged recombinant mouse serum albumin improved the health span and lifespan of mice. These findings suggest that neutral plasma exchange or plasma albumin could be therapeutic. Imran et al (Iram et al., 2022, p.17) found that young cerebrospinal fluid restores oligodendrogenesis and memory in aged mice via serum response factor, and alternatively, fibroblast growth factor 17. Our subjects were given approximately 5 mg of plasma concentrate pellet dissolved in 1 ml of saline weekly for 10 consecutive weeks. This is an insignificant amount of volumetric dilution, considering that the average 70 kg male has 3000 ml of plasma and that the average healthy adult produces between 400 and 2000 ml per day. Our study lends credence to the notion that there are youth‐promoting factors in the secretome of umbilical cord plasma. This conclusion has also been reached by other researchers that have provided treatment with stem cells, which, again, do not work by plasma dilution but primarily by providing humoral factors and changing the microenvironment of cells and tissues. While there may be youth‐promoting microvesicles or humoral factors that are at work, we do not want to rule out the possibility that it is “young and undamaged” albumin that leads to the improvements noted, especially in the light of recent evidence for such a mechanism.

In conclusion, this first human epigenetic clock study of plasma concentrate treatments revealed age‐reversal effects according to a well‐established DNA methylation‐based estimator of morbidity and mortality risk. Traditional clinical biomarkers support the view that the treatment had beneficial effects, and no significant adverse effects were reported. Future, placebo‐controlled replication studies are warranted with a larger number of participants over a longer study period, which our laboratory has undertaken to pursue.

## 4 EXPERIMENTAL PROCEDURES

### 4.1 Ethics

Informed consent was obtained from all subjects, in compliance with Institutional Review Board‐approved protocols and in accordance with the Declaration of Helsinki. Ethics committee approval identifiers were IRCM Study Number IRCM‐2018‐175 and Protocol BH‐PC‐104.

### 4.2 Study enrollment

Participants were obtained from among existing patients of the Study Physician, and individuals known to the Principal Investigator. Twenty (20) men and women from the ages 65 to 95 were selected, after meeting the Study Protocol's Inclusion and Exclusion Criteria. Key exclusion criteria included (1) Female patients who have not reached menopause; (2) patients currently or within the last 3 years receiving radiation or chemotherapy treatment for cancer; (3) patients with untreated or uncontrolled metastasis; (4) patients with immunocompromised conditions; and (5) patients not meeting a general physical and blood chemistry screening administered by the study's physician anytime within the past year. The participants were considered to be in “normal health for their ages.” A few had type‐2 diabetes and were taking insulin on a daily basis. Many were on medications for conditions such as hypertension and high cholesterol. One woman had early onset dementia and rheumatoid arthritis. None of the participants exhibited other severe conditions. Two patients dropped out from the trial for non‐health‐related reasons, resulting in 18 subjects for whom all treatments, blood samples, and examinations were completed.

### 4.3 Human umbilical cord blood plasma concentrate treatment

The treatments consisted of an intramuscular injection of human umbilical cord blood plasma (hUCBP) concentrate obtained pursuant to mutually agreed upon specifications with the Institute of Regenerative and Cellular Medicine (IRCM, Santa Monica, CA). Units of hUCBP were obtained from a registered cord blood bank, tested and found to be negative for Infectious Disease Markers: HIV 1/2, HIV p24: HTLV – I, anti‐HBc, HBs antigen, anti HCV, CMV, and RPR according to the providers testing SOPs. In addition, all source material was successfully quarantined and found to be acceptable (pass) showing no microorganism growth (acceptable micro). The plasma was handled inside an ISO‐5 environment, and a series of centrifugations were done to remove cellular components, cellular debris, and protein contaminants. Post‐centrifugation components included, but were not limited to, microvesicles, enzymes, and proteins. Measurement of EVs contained in the isolate ranged in size from 50 to 150 nm. After serial centrifugation steps were completed, the final, concentrated “pellet” (weighing approximately 5 mg) was reconstituted in 1 ml of sterile 0.9% sodium chloride injection USP, then filter sterilized using a 0.22 μm syringe filter, and kept at −80° C until use. It is important to note that no additional or external chemicals were introduced to the sample at any time point during the isolation processing, until the final step that involved using saline to reconstitute the pellet. The entire components of a 100 ml of hUCBP were concentrated to 1 ml volume, processed as described above, and were subsequently injected by syringe intramuscularly, generally in the deltoid, by the Study Physician once a week, for a period of 10 weeks.

### 4.4 Clinical biomarkers

In total, we measured 78 clinical biomarkers for each participant before and after the treatment. For analyses of lymphocytes and red blood cells, a complete blood count test (CBC) was performed. A CBC with differential and platelet count is a common screening test for multiple diseases, including anemia, leukemia, and inflammation. The methodology used for this test is electronic cell sizing or cytometry, and generally is performed pursuant to a CLIA‐approved automated system. A blood chemistry panel was also performed that includes many analytes routinely ordered to determine general health using serum or blood. These included measures of glucose and calcium, kidney health based on blood urea nitrogen (BUN) and creatinine, tests of liver function with alkaline phosphatase, levels of electrolytes including sodium, potassium, and carbon dioxide, as well as protein measures of albumin and total protein. Many of these factors also are known to change with age and have been used to develop multivariate models that predict morbidity and mortality (Levine et al., 2018).

### 4.5 DNA methylation and epigenetic clock analysis

For each participant, peripheral whole blood samples were collected, and we performed bisulfite conversion using the Zymo EZ DNA Methylation Kit (Zymo Research, Orange, CA, USA) as well as subsequent hybridization of the HumanMethylationEPIC Bead Chip (Illumina, San Diego, CA), and scanning (iScan, Illumina) according to the manufacturer's protocols by applying standard settings. Raw methylation signal intensities were retrieved using the function *read.metharray.exp* of the *minfi* R package, followed by linear dye bias correction and noob background correction to account for technical variation in background fluorescence signal (Aryee et al., 2014). β‐values were used in all analyses. Specifically, the β‐value was calculated from the intensity of the methylated and unmethylated sites, as the ratio of fluorescent signals. In total, 866,238 probes were included in downstream analyses.

We included six different DNA methylation‐based aging biomarkers or epigenetic clocks in this study. This included DNAmGrimAge (Lu, Quach, et al., 2019) and DNAmPhenoAge (Levine et al., 2018), which are morbidity and mortality predictors, as well as chronological age estimators such as the pan‐tissue clock (Horvath, 2013), Hannum clock, and the Skin & Blood Age clock (Horvath et al., 2018). Utilizing our online DNA Methylation Age Calculator (http://dnamage.genetics.ucla.edu/new), we calculated DNA methylation‐based ages (DNAmAge) and age‐adjusted versions referred to as epigenetic age acceleration (AgeAccel). All measures of epigenetic age acceleration were defined as raw residuals resulting DNAmAge estimates on the chronological age. By definition, these residuals are not correlated with age (Pearson correlation *r* = 0). To briefly describe each, the pan tissue clock was calculated using a linear combination of 353 CpGs that have previously been shown to predict chronological age in multiple tissues (Horvath, 2013); Hannum blood‐based clock was calculated using a linear combination of 71 CpGs to predict chronological age in the blood (Hannum et al., 2013); DNAmSkinClockAge was calculated on the basis of 391 CpGs (Horvath et al., 2018); DNAmTL was calculated as the surrogate marker of telomere length; DNAmPhenoAge was calculated based on 513 CpGs (Levine et al., 2018); and DNAmGrimAge clock was calculated from 1030 CpGs (Lu, Quach, et al., 2019). The mortality risk estimator DNAmGrimAge can be interpreted as a linear combination of 7 DNAm‐based estimates of plasma protein and a DNAm‐based estimator of smoking pack‐years (Lu, Quach, et al., 2019).

White blood cell composition was imputed for each study participant and included in downstream analyses: CD4+ T, naive CD8+ T, exhausted cytotoxic CD8+ T cells (defined as CD8 positive CD28 negative CD45R negative, CD8 + CD28‐CD45RA‐), plasmablasts, monocytes, natural killer cells, and granulocytes. Naive CD8+ T, exhausted cytotoxic CD8+ T cells, and plasmablasts were calculated as described in reference (Horvath & Levine, 2015). The remaining cell types were imputed using the Houseman method (Houseman et al., 2012).

### 4.6 Epigenome‐wide association study

The global mean methylation level was calculated by averaging genome‐wide methylation levels across all 866,238 CpG sites. For functional enrichment analyses, we focused on the set of top 500 CpGs that were hypermethylated after the treatment and the top 500 CpGs that were hypomethylated after the treatment. To investigate the relationship between top CpGs associated with the treatment and known aging‐related genes, we first overlapped our results with the GenAge database, a curated database of over 300 genes related to human aging (Tacutu et al., 2018). We conducted hypergeometric tests to test whether treatment‐related CpGs were randomly distributed across the genome or were more likely to be found in specific genomic regions (CpG islands, shores, shelves, or open sea area). CpGs were annotated using *IlluminaHumanMethylationEPICanno.ilm10b4.hg19* R package.

In addition, we calculated genomic region‐specific mean methylation levels for CpGs within transcription start sites (TSS1500, TSS200), untranslated regions (5'UTR, 3'UTR), 1st Exon, and gene body. The association between treatment and global/genomic region‐specific mean methylation levels was calculated using paired t‐test. We identified CpG probes that are changed after treatment by conducting paired t‐test for each probe separately. We accounted for multiple testing via the false discovery rate (FDR)‐adjusted *p*‐values. To investigate the potential bias caused by cellular heterogeneity, we also conducted a sensitivity analysis by adjusting for monocytes in the model.

### 4.7 GREAT enrichment analysis

To uncover biological processes potentially linked to treatment‐related CpGs, we then employed the Genomic Regions Enrichment of Annotations Tool (GREAT) (McLean et al., 2010) to identify functional annotations associated with genes that are proximal to treatment‐related CpGs. The extension of gene regulatory regions was set at 50 kb, and the other options were based on default settings. By specifying the background, GREAT analysis performed genomic region‐based hypergeometric analysis, not confounded by gene sizes and uneven gene coverage. Utilizing the GREAT method, we conducted the gene ontology (GO) biological process, molecular function, and cellular component pathway enrichment analysis, Molecular Signatures Database (MSigDB) pathway enrichment analysis, disease ontology enrichment analysis, and human phenotype enrichment analysis. GO terms were simplified for easier interpretation by calculating the similarity of GO terms and removing those highly similar terms using the *rrvgo* method.

### 4.8 Methylation‐Based measure of Shannon entropy

As a measure for a well‐functioning epigenetic maintenance system that maintains genomic stability, we calculated the Shannon entropy of the methylome based on all EPIC array probes. The formula of Shannon entropy is

$$\text{Entropy}=\frac{1}{N\times \log_{2}\left(\frac{1}{2}\right)}\times \sum_{i=1}^{N}\left[\beta_{i}\times \log_{2}\left(\beta_{i}\right)+\left(1-\beta_{i}\right)\times \log_{2}\left(1-\beta_{i}\right)\right]$$

where $\beta_{i}$ represents the methylation beta value for the i^th^ probe (CpG site) in the array, N represents the total number of probes included in the formula (Hannum et al., 2013). An increase in entropy means that the methylome becomes less predictable across the population of cells, that is, when the methylation fractions (beta values) tend toward 50%.

### 4.9 Statistical analysis

All analyses were conducted using R v4.0.5. We used pairwise Pearson correlations to assess the relations between chronological age, clinical biomarkers, cell types, and DNAm AgeAccel values. Paired t‐test was used to examine the changes of clinical biomarkers, cell types, DNAm AgeAccel, and methylation for each probe after treatment compared to baseline levels before treatment.

## AUTHOR CONTRIBUTIONS

The Principal Investigator of the Study was JC and the Study Physician was JAS. JC and REC shared equally in the design and implementation of the study. JC and QY shared equally in the writing of this paper. QY, ATL, SH carried out the statistical analysis. All authors contributed to the writing and the interpretation of the results.

## CONFLICT OF INTEREST

James Clement is a founder of Betterhumans Inc., a nonprofit medical research organization with offices in Gainesville, FL and Tyler, Texas focused on slowing and reversing the detrimental effects of aging. Robert T. Brooke and Steve Horvath are founders of the nonprofit Epigenetic Clock Development Foundation. The Clock foundation licenses inventions and patents surrounding epigenetic clocks from UC Regents. These patents list Steve Horvath as inventor.

## Supporting information


Figure S1

Table S1

## Data Availability

Raw and processed DNA methylation data are available from the Gene Expression Omnibus (GEO) under accession number GSE210245.
