# Supplementary material for: Umbilical cord plasma concentrate has beneficial effects on DNA methylation GrimAge and human clinical biomarkers
Source: Aging Cell. 2022 Sep 2;21(10):e13696. doi: 10.1111/acel.13696 (PMC9577957; doi:10.1111/acel.13696)
Supplement: Supplementary file 1 — Figure S1 [file ACEL-21-e13696-s002.docx]

**SUPPLEMENTARY FIGURES**

**Supplementary Figure S1. Pairwise correlations between selected clinical biomarkers and epigenetic age acceleration**. Apart from clinical biomarkers, the figure includes epigenetic age acceleration of the pan tissue clock (AgeAccelHorvath) and chronological age. Only clinical biomarkers significantly changed after the treatment were included in the figure. The shades of color (blue, white, and red) visualize Pearson correlation values from ‐1 to 1. Each square reports a Pearson correlation coefficient.

**Supplementary Figure S2. Pairwise correlations of methylation-based biomarkers and chronological age.** The heatmap color‐codes the pairwise Pearson correlations. Age represents the chronological age. AgeAccelHorvath, AgeAccelHannum, AgeAccelSkinBlood, AgeAccelTL, AgeAccelPheno, and AgeAccelGrim represent measures of epigenetic age derived from the Horvath pan tissue clock, the Hannum clock, the SkinBlood clock, the surrogate markers of telomere length, the PhenoAge clock, and the GrimAge clock, respectively. The shades of color (blue, white, and red) visualize correlation values from ‐1 to 1. Each square reports a Pearson correlation coefficient.

**Supplementary Figure S3. Paired boxplots of the epigenetic age acceleration (AgeAccel) effects due to the treatment.** AgeAccel is defined as the raw residuals derived from regression models of DNAm ages on chronological age. The y-axis shows the age acceleration rate for each DNAm age estimator tested. Panel a to e showed changes of AgeAccel derived from Horvath’s pan-tissue epigenetic age (referred to as AgeAccelHorvath), Hannum’s blood-based DNAm age (AgeAccelHannum), skin and blood clock (AgeAccelSkinBlood), DNAm the surrogate markers of telomere length (AgeAccelTL), and DNAmPhenoAge (AgeAccelPheno), respectively. P-values were derived from paired t-tests.

**Supplementary Figure S4. Paired boxplots of methylation-based estimates of blood cell composition.** Panel a to g showed changes of naïve CD8 cells (CD8.naive), exhausted CD8+ cells (CD8pCD28nCD45RAn), Plasma Blasts, CD4 T cells (CD4T), natural killer cells (NK), monocytes (Mono), and granulocytes (Gran), respectively. P-values were derived from paired t-tests.

**Supplementary Figure S5. Telomere Length and mtDNA Copy Number.** The Left and right panels showed changes of telomere length and mtDNA copy number, respectively. P-values were derived from paired t-tests.

**Supplementary Figure S6.** QQ-plot (left panel) shows the observed minus log10-transformed P values obtained from paired t-tests against the theoretical null distribution quantiles. Histogram of p-values (right panel) showed the distribution of raw p-values.

**Supplementary Figure S7.** Gene set enrichment analysis of significant CpGs related to the treatment. The gene-level enrichment was done using GREAT analysis. The input was top 500 hypermethylated probes and top 500 hypomethylated probes. **(a)** Disease ontology. **(b)** Human phenotype.


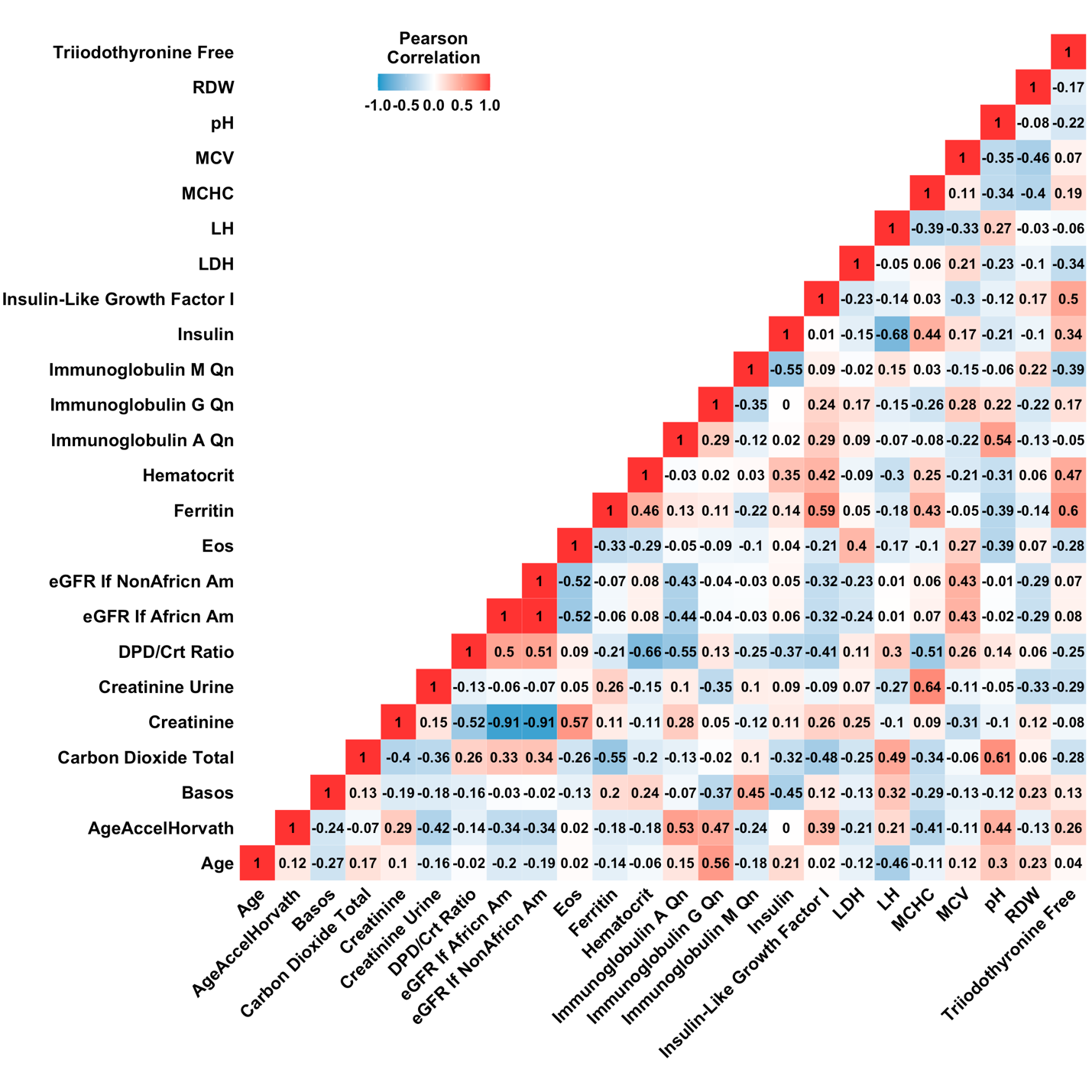


**Supplementary Figure S1. Pairwise correlations between selected clinical biomarkers and epigenetic age acceleration.** Apart from clinical biomarkers, the figure includes epigenetic age acceleration of the pan tissue clock (AgeAccelHorvath) and chronological age. Only clinical biomarkers significantly changed after the treatment were included in the figure. The shades of color (blue, white, and red) visualize Pearson correlation values from ‐1 to 1. Each square reports a Pearson correlation coefficient.


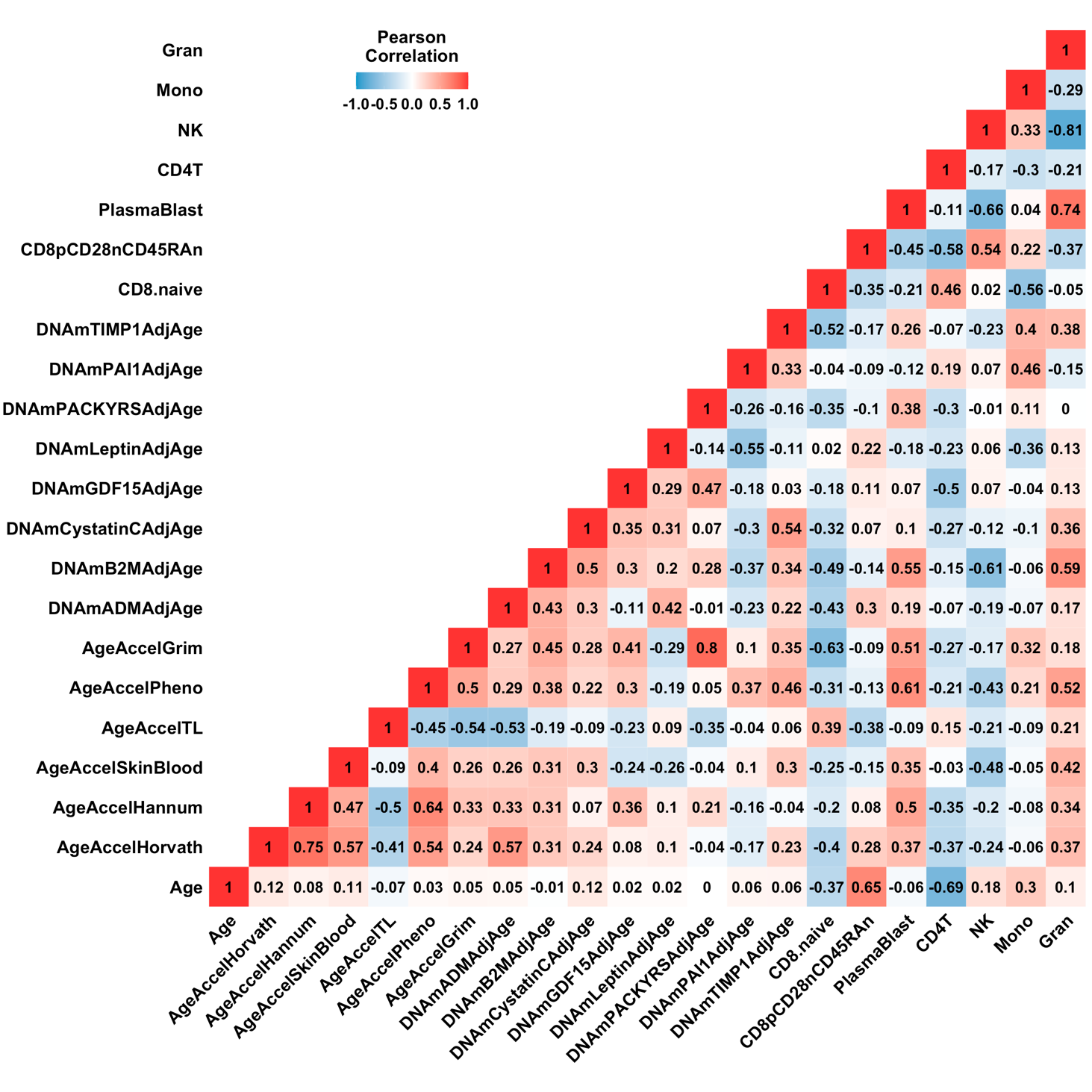


**Supplementary Figure S2. Pairwise correlations of methylation-based biomarkers and chronological age.** The heatmap color‐codes the pairwise Pearson correlations. Age represents the chronological age. AgeAccelHorvath, AgeAccelHannum, AgeAccelSkinBlood, AgeAccelTL, AgeAccelPheno, and AgeAccelGrim represent measures of epigenetic age derived from the Horvath pan tissue clock, the Hannum clock, the SkinBlood clock, the surrogate markers of telomere length, the PhenoAge clock, and the GrimAge clock, respectively. The shades of color (blue, white, and red) visualize correlation values from ‐1 to 1. Each square reports a Pearson correlation coefficient.


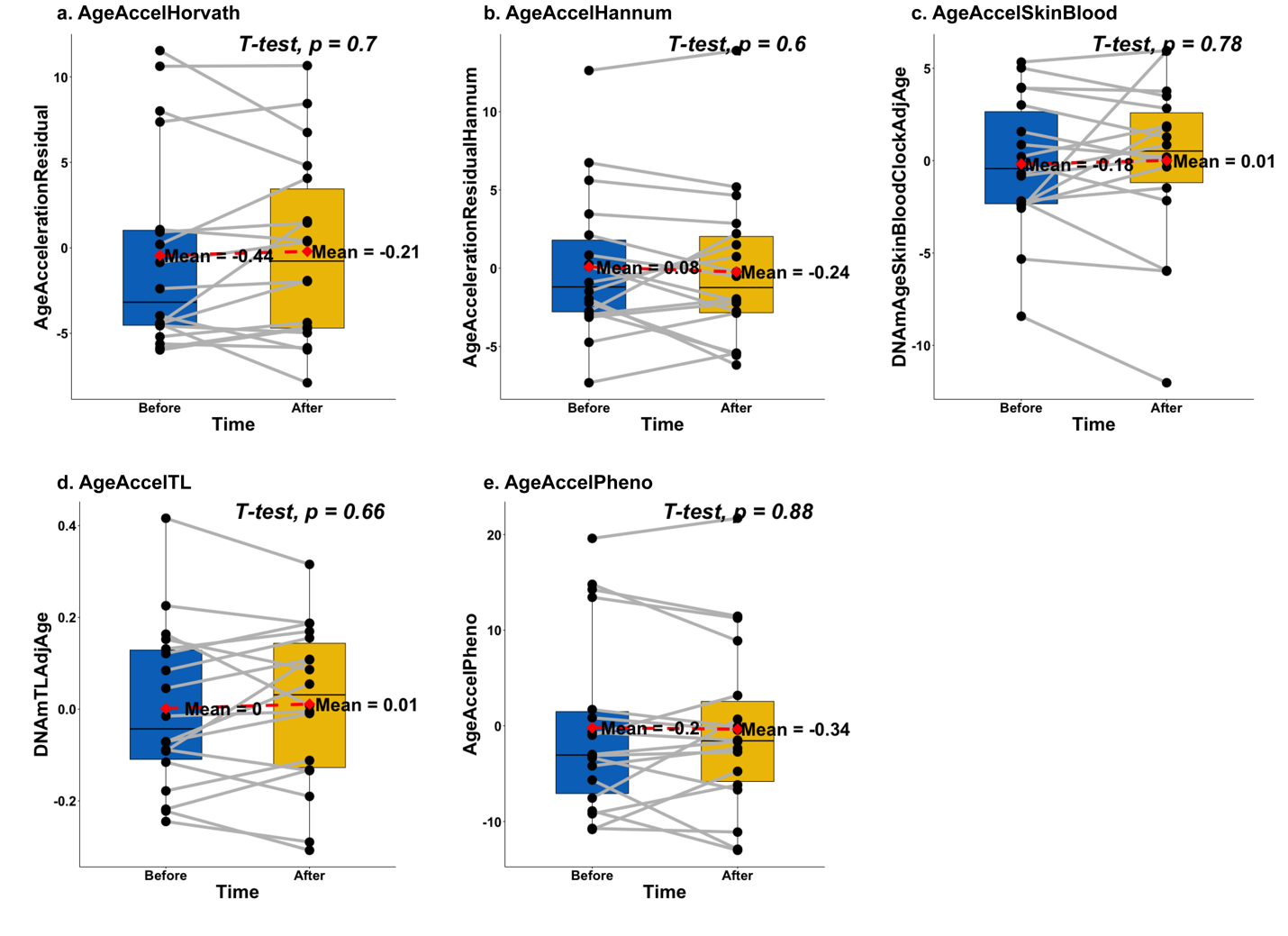


**Supplementary Figure S3. Paired boxplots of the epigenetic age acceleration (AgeAccel) effects due to the treatment.** AgeAccel is defined as the raw residuals derived from regression models of DNAm ages on chronological age. The y-axis shows the age acceleration rate for each DNAm age estimator tested. Panel a to e showed changes of AgeAccel derived from Horvath’s pan-tissue epigenetic age (referred to as AgeAccelHorvath), Hannum’s blood-based DNAm age (AgeAccelHannum), skin and blood clock (AgeAccelSkinBlood), DNAm the surrogate markers of telomere length (AgeAccelTL), and DNAmPhenoAge (AgeAccelPheno), respectively. P-values were derived from paired t-tests.

**
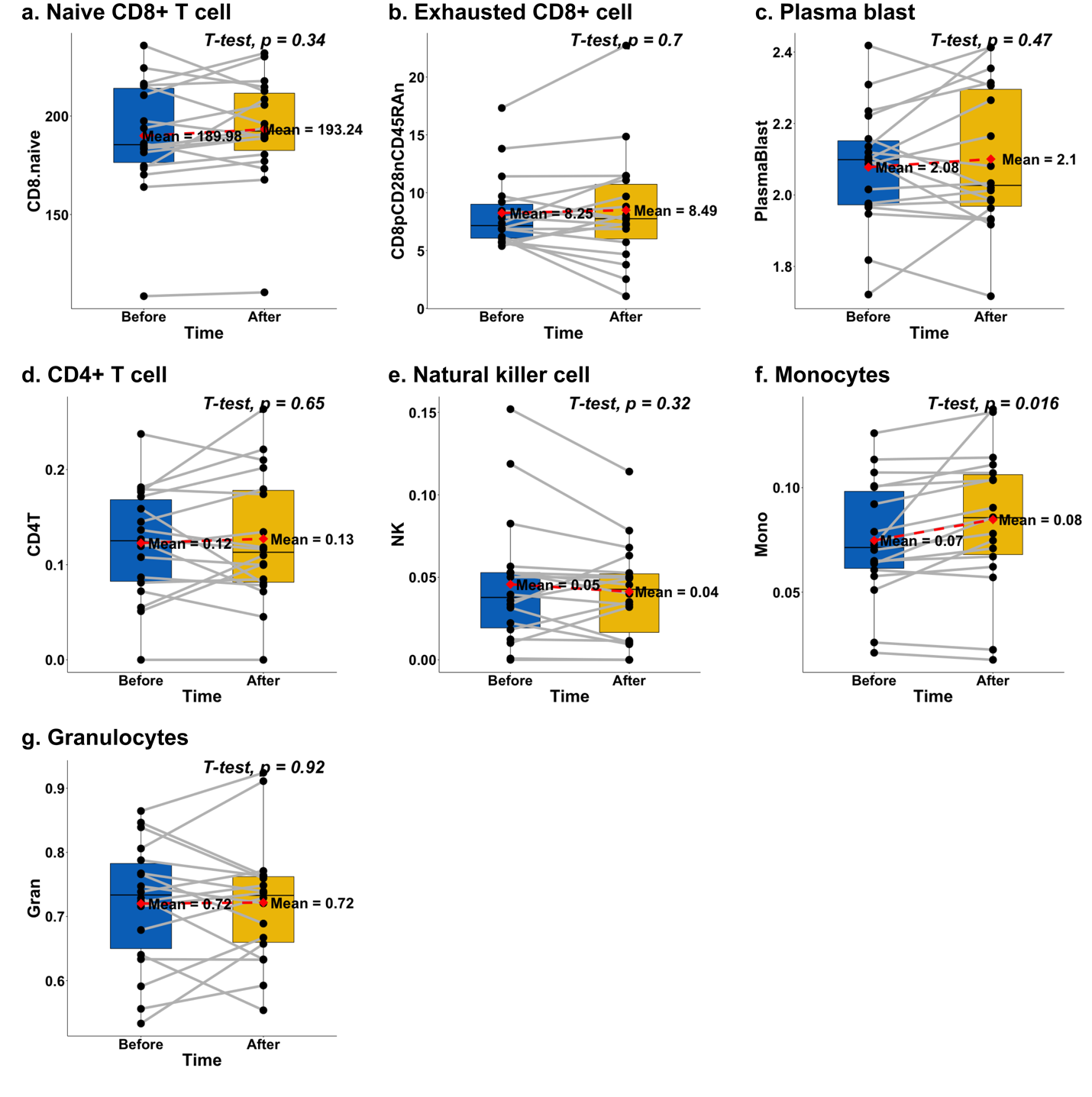
Supplementary Figure S4. Paired boxplots of methylation-based estimates of blood cell composition.** Panel a to g showed changes of naïve CD8 cells (CD8.naive), exhausted CD8+ cells (CD8pCD28nCD45RAn), Plasma Blasts, CD4 T cells (CD4T), natural killer cells (NK), monocytes (Mono), and granulocytes (Gran), respectively. P-values were derived from paired t-tests.


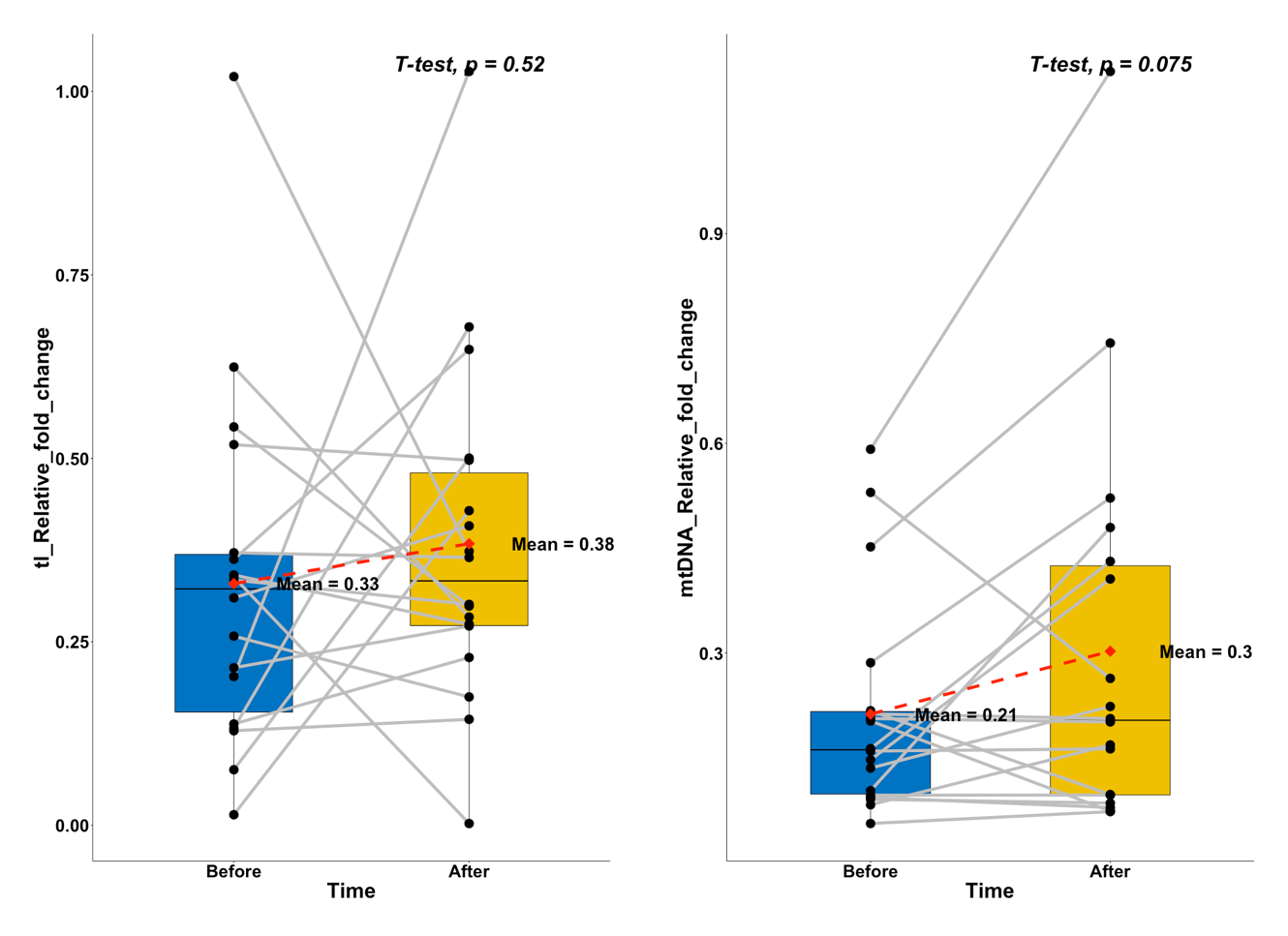


**Supplementary Figure S5. Telomere Length and mtDNA Copy Number.** The Left and right panels showed changes of telomere length and mtDNA copy number, respectively. P-values were derived from paired t-tests.

**
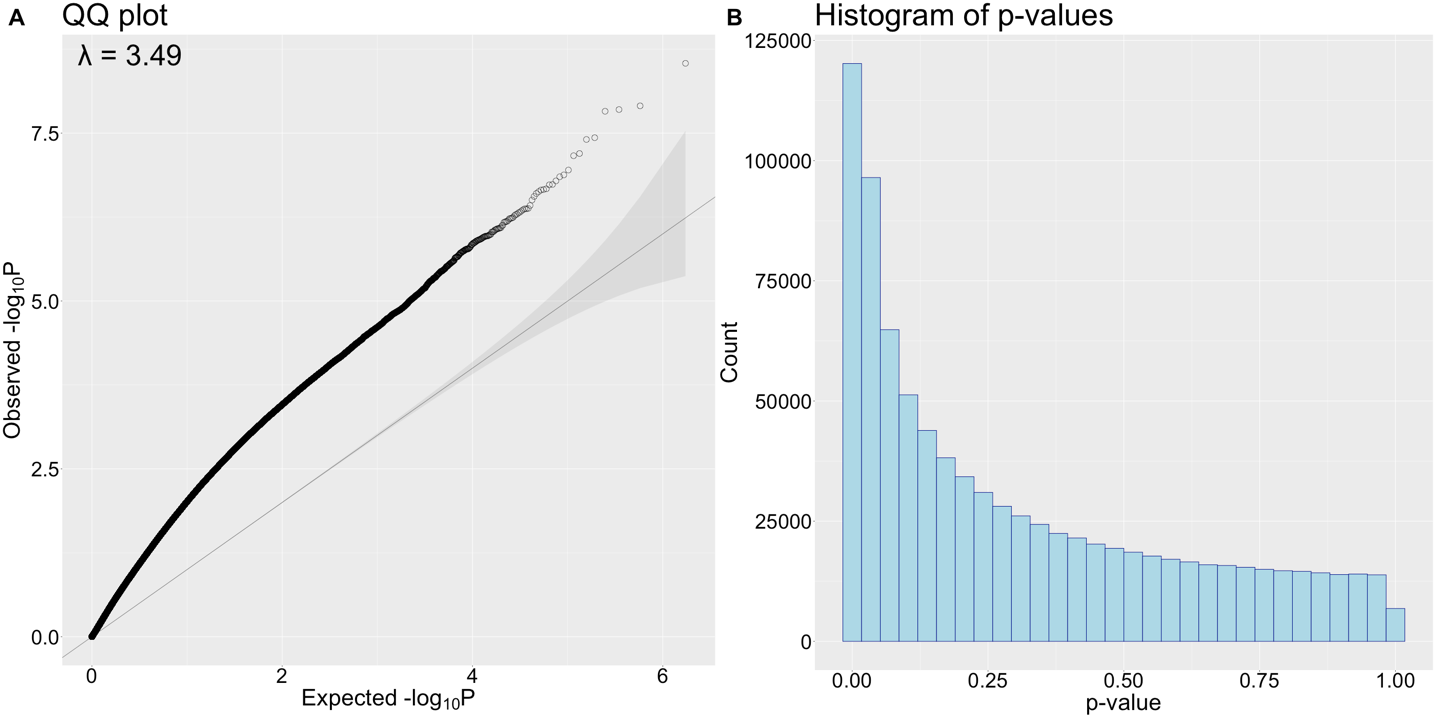
**

**Supplementary Figure S6.** QQ-plot (left panel) shows the observed minus log_10_-transformed P values obtained from paired t-tests against the theoretical null distribution quantiles. Histogram of p-values (right panel) showed the distribution of raw p-values.


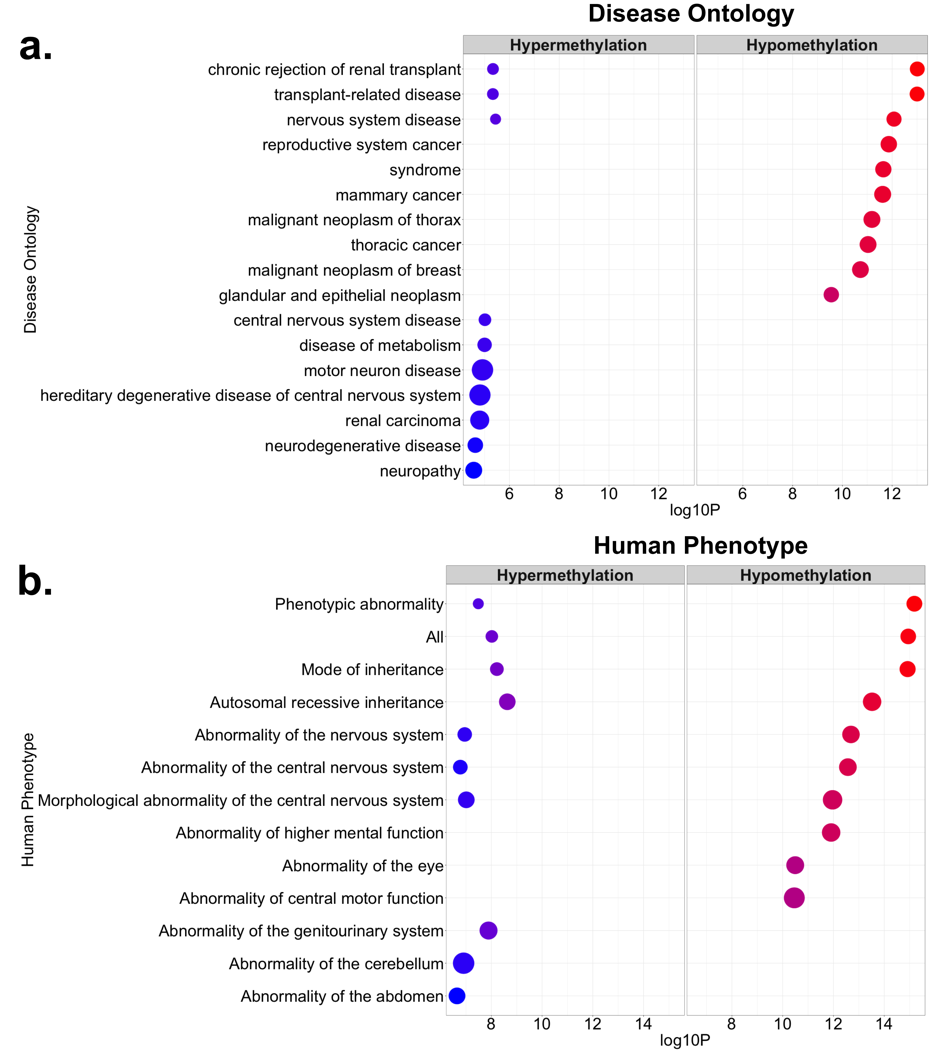
**Supplementary Figure S7.** Gene set enrichment analysis of significant CpGs related to the treatment. The gene-level enrichment was done using GREAT analysis. The input was top 500 hypermethylated probes and top 500 hypomethylated probes. **(a)** Disease ontology. **(b)** Human phenotype.
